# Women's education, contraception use, and high-risk fertility behavior: A cross-sectional analysis of the demographic and health survey in Ethiopia

**DOI:** 10.3389/fgwh.2023.1071461

**Published:** 2023-03-01

**Authors:** Berhanu Teshome Woldeamanuel, Getachew Tilahun Gessese, Takele Gezahegn Demie, Simegnew Handebo, Tolesa Diriba Biratu

**Affiliations:** Department of Epidemiology and Biostatistics, School of Public Health, St. Paul's Hospital Millennium Medical College, Addis Ababa, Ethiopia

**Keywords:** demographic and health survey, high-risk fertility behavior, women education, contraception use, Ethiopia

## Abstract

**Background:**

High-risk fertility behavior (HRFB) among women is the main factor in a wide range of detrimental effects on both the mother's and the child's health, which has an impact on both the mother's and the child's survival. Early childbearing is associated with a higher number of live births and may result in poorer maternal, baby, and child health outcomes. Infant and child mortality are also linked to short birth intervals and higher birth order. Thus, this study aims to examine the link between women's education, contraception use, and high-risk fertility behavior in Ethiopia.

**Methods:**

Data was drawn from the 2019 Ethiopian Interim Demographic and Health Survey. The analysis covered a total of 5,846 women. The effect of predictor variables on HRFB was quantified using multivariable logistic regression analysis. At a 95% CI of the odds ratio excluding one, a significant association between the HRFB and predictor variables was observed.

**Results:**

About 72.8% (95% CI 71.6%–73.9%) of women experience high-risk fertility behaviors. Of these, 32% experience single high-risk fertility behavior, and 40.8% experience multiple high-risk fertility behaviors. Of those who experience high-risk fertility behaviors, 58.7% have birth orders of more than three, 22.4% have short birth intervals (less than 24 months); 35.1% are old (over 34 years old); and 1.6% are young (less than 18 years old). Women with no education (AOR = 4.31; 95% CI: 2.09, 8.89) and primary education only (2.71; AOR = 2.71; 95% CI: 1.63, 4.50) are more likely to engage in high-risk fertility behaviors than women with a higher level of education. Every additional year of schooling reduces the odds of high-risk fertility behavior by 6% (AOR = 0.94; 95% CI: 0.89, 0.98). The use of modern contraception (AOR = 0.74; 95% CI: 0.622, 0.879) and knowledge of modern contraception methods (AOR = 0.80; 95% CI: 0.66, 0.96) reduce the risk of HRFB.

**Conclusions:**

Primary education and a lack of education significantly raise the risk of HRFB. However, in Ethiopia, the risk of experiencing HRFB is reduced through modern contraceptive methods, awareness of modern contraceptive methods, and years of education. All initiatives to decrease maternal and newborn mortalities by reducing the risk of HRFB should educate women and encourage them to use modern contraception.

## Introduction

Women's high-risk fertility behavior (HRFB), including behaviors such as early (under age 18) or late (over age 34) childbearing, a higher number of live births (more than three children), and close birth spacing (at most, 24 months between births), is the primary root of abundant detrimental effects on both the mother's and the child's health (1). The risk is elevated for children born to a mother who carries a mix of these risk factors (2). Despite a 38% reduction in global maternal and child mortality rates from 2000 to 2017, poor countries account for 94% of maternal deaths (3). Sub-Saharan Africa accounts for two-thirds of the global maternal death estimate (4). The vast majority of maternal deaths in impoverished nations are caused by risks associated with pregnancy and childbirth and inadequate health service performance.

Every day in 2019, over 810 women died all over the world from preventable maternal and childbirth-related causes (5). The third Sustainable Development Goal (SDG-3) will continue to prioritize preventing preventable maternal and child deaths, bringing down neonatal mortality to 12 or fewer deaths per 1,000 live births, under-five mortality to 25 or fewer deaths per 1,000 live births, and worldwide maternal mortality to fewer than 70 deaths per 100,000 live births (6). According to the interim Demographic and Health Survey, Ethiopia's infant mortality rate was 47 deaths per 1,000 live births in 2019, while under-five mortality was 59 deaths per 1,000 live births. However, neonatal mortality increased in 2016, rising from 29 to 33 fatalities per 1,000 live births (7). Ethiopia's maternal and infant death rates are still alarming.

Early childbearing is associated with more children being born, which is associated with poorer maternal, newborn, and child health outcomes (8). The mortality of infants and children is similarly correlated with short birth intervals (24 months) (9, 10) and higher birth order (11). The short birth-to-pregnancy interval is one of the significant factors that influence infant and under-five mortalities. Prior research has demonstrated that a short-preceding birth interval has a destructive effect on early infancy and child mortality (12); in cases where the previous child is still living, child mortality is more pronounced (13).

If vaginal delivery occurs after cesarean delivery, there is a higher risk of untimely membrane rupture, uteroplacental hemorrhage problems, and uterine rupture when there are short previous intervals (14, 15). Short intervals may cause nutritional depletion in the mother, and inadequate recovery time between pregnancies could worsen the mother's nutritional status.

The impacts of maternal age include the likelihood of having more children for women who start having children earlier in life, which is associated with poorer outcomes for maternal, newborn, and child health (8, 16). This can account for the social disadvantage faced by young women as well as other social comorbidities, such as childhood sexual abuse, family violence, community violence, sexual assault, and depression. Preterm birth and stillbirth have also been linked to childbearing at an older age (35 or older) (17–20). It is widely acknowledged that the natural effects of aging have an impact on older women's reproductive health and ability to have children. Young teenage mothers are not physiologically and reproductively mature, and this may upsurge the possibility of complications throughout pregnancy and delivery as well as insufficient weight gain. Teenage mothers may also compete for nutrients with the fetus (21, 22), and psychological immaturity may also upset the child's care. Women with higher parities are also more at risk of poor maternal outcomes and newborn and child mortality (23, 24). The cumulative effects of delivery and breastfeeding may jeopardize women's health in high-parity situations. The association between education and fertility draws on the indication that women's decisions about having children and other lifestyle choices are linked (25). Higher levels of education may result in greater engagement employee (26). Education also improves mothers' access to and awareness of contraception, which in turn reduces fertility (27). Extensive studies support a significant association between women's education levels and higher usage of contraceptive methods (28–30). According to a study conducted in Egypt (30), the enablement procedure with advances in educating women is in effect to control their fertility behavior, resulting in reduced fertility. Education empowers the vulnerable in society by providing them with more options (31). It has not been investigated how women's empowerment through education affects fertility.

The use of contraceptives affects women's and children's health and survival by lowering the number of births and the proportion of births that fall into the category of high-risk behavior by avoiding births at both significantly young and old ages, thus leading to overall reductions in fertility risk. Ahmed et al. (2012) estimated that in 2008, contraceptive use averted around 44% of maternal deaths (32). Cleland and colleagues (2012) discovered that contraception use also prevented an additional 3.7% of maternal deaths in 2008, in addition to lowering fertility (23). It is widely believed that by delaying early pregnancy using modern contraceptive methods, several thousand births at a young age could be avoided. Family planning initiatives are drivers of fertility decline and reduce both desired and undesired fertility (25).

Women who have acquired better education are more likely to make decisions about their fertility desires. As a result, there is a nexus between women's education levels, women's contraception use, and fertility behavior (33). Despite various studies concentrating on the drivers of high-risk fertility behavior, the quantitative influence of women's education and contraceptive use on high-risk fertility behavior in Ethiopia has yet to be measured. The objective of this study is to investigate the link between women's education, contraception use, and high-risk fertility behavior. We sought to answer the question of whether the number of years of education a woman has had is associated with a significant decline in high-risk fertility behavior in Ethiopia.

## Methods

### Study design and data source

This study analyzes data from the 2019 Ethiopia Mini Demographic and Health Survey (EMDHS), a cross-sectional survey conducted in Ethiopia in 2019. Interviews of 8,855 eligible women were taken, with retrospective questions spanning five years before the survey.

### Sampling approach and study population

The 2019 Ethiopian mini-DHS used a two-stage sample design. The first step was to choose a sample of clusters made up of enumeration areas (EAs) generated for the 2019 Ethiopian Population and Housing Census (EPHC). A total of 305 EAs were chosen in the first stage. Implicit stratification and proportional allocation were achieved at each of the lower administrative levels by sorting the sampling frame within each sampling stratum before sample selection, according to administrative units at various levels, and selecting a probability proportional to size at the first stage of sampling. From eight regions, 25 EAs were selected, and 35 EAs were chosen from each of the three essential regions (Amhara; Oromia; and the Southern Nations, Nationalities, and Peoples' Region (SNNPR)) to make certain that survey precision became comparable across regions. A specified number of 30 households per cluster were selected in the second step of the selection process, with an equal likelihood of systematic selection from the newly formed household listing. The current study involves 5,846 women aged 15–49 years old (7).

### Outcome and predictor variables

High-risk fertility behavior was defined as births at a significantly young age (less than 18 years), births at a significantly old age (> 34 years), a short birth-to-pregnancy interval (less than 24 months), and a high birth order (4 births or more). The definition of HRFB in this study was adopted from the Measure DHS and Ethiopian Demographic and Health Survey (1, 7). The explanatory variables included women's highest level of education, place of residence, geographical region, access to TV or radio, household wealth quintile, marital status, religion, contraceptive use, and knowledge of modern contraception. In the DHS, the highest level of education was measured by the number of years of schooling.

### Data analysis and software

The statistical software IBM SPSS® Statistics 25.0 was used to conduct data analysis. To adjust for the differential probability of sampling and non-response, sample weights were used. Both the bivariate and multivariable binary logistic regression models were used to model HRFB and associated factors. Variables that were significant at the 20% level in the bivariate analysis were incorporated into the multivariable analysis. The test of model adequacy was checked using the Hosmer and Lemeshow test (HLT) and the Likelihood ratio test (LRT). Furthermore, the significant test of LRT was considered a good fit for the model.

## Results

### Characteristics of the respondents

This study involves 5,846 women aged 15–49 who gave birth in the five years before the survey from the 2019 EMDHS dataset. Over half (54.2%) of the women were uneducated. The majority (71.8%) of the women were rural residents, and 60.2% had no access to TV or radio. Approximately two-thirds (67.6%) of the women did not use modern contraceptives, and 80.7% of them knew about modern contraception methods. Most of the women (86.9%) were married or living with their partners; 35.3% were Orthodox Christians, and 42.8% were Muslims by religion (Table 1).

**Table 1:** Socio-demographic characteristics, *n* = 5846.

Variables	Categories	*n*	%
**Region**	Tigray	504	8.6%
	Afar	500	8.6%
	Amhara	643	11.0%
	Oromia	708	12.1%
	Somali	430	7.4%
	Benishangul-gumuz	523	8.9%
	SNNPR	690	11.8%
	Gambela	529	9.0%
	Harari	476	8.1%
	Dire Dawa	469	8.0%
	Addis Ababa	374	6.4%
**Residence**	Urban	1648	28.2%
	Rural	4198	71.8%
**Highest education level**	No education	3176	54.3%
	Primary	1811	31.0%
	Secondary	529	9.0%
	Higher	330	5.6%
**Head of Household**	Husband/Other	4356	74.5%
	Respondent	1490	25.5%
**Wealth quintile**	Poorest	1515	25.9%
	Poorer	968	16.6%
	Middle	893	15.3%
	Richer	889	15.2%
	Richest	1581	27.0%
**Access to TV or Radio**	No	3476	60.2%
	Yes	2299	39.8%
**Marital status**	Single	59	1.0%
	Married/living together	5079	86.9%
	Others	708	12.1%
**Religion**	Orthodox	2063	35.3%
	Protestant	1165	19.9%
	Muslim	2500	42.8%
	Others	118	2.0%
**Contraceptive use**	No	3953	67.6%
	Yes	1893	32.4%
**Number of antenatal visits during pregnancy**	No antenatal visits	1044	26.2%
	1	141	3.5%
	2	353	8.9%
	3	768	19.3%
	4+	1656	41.7%
	8+	120	3.1%
**Knowledge of modern contraceptive methods**	No	1129	19.3%
	Yes	4717	80.7%

### High-risk fertility behavior

**Figure 1:**
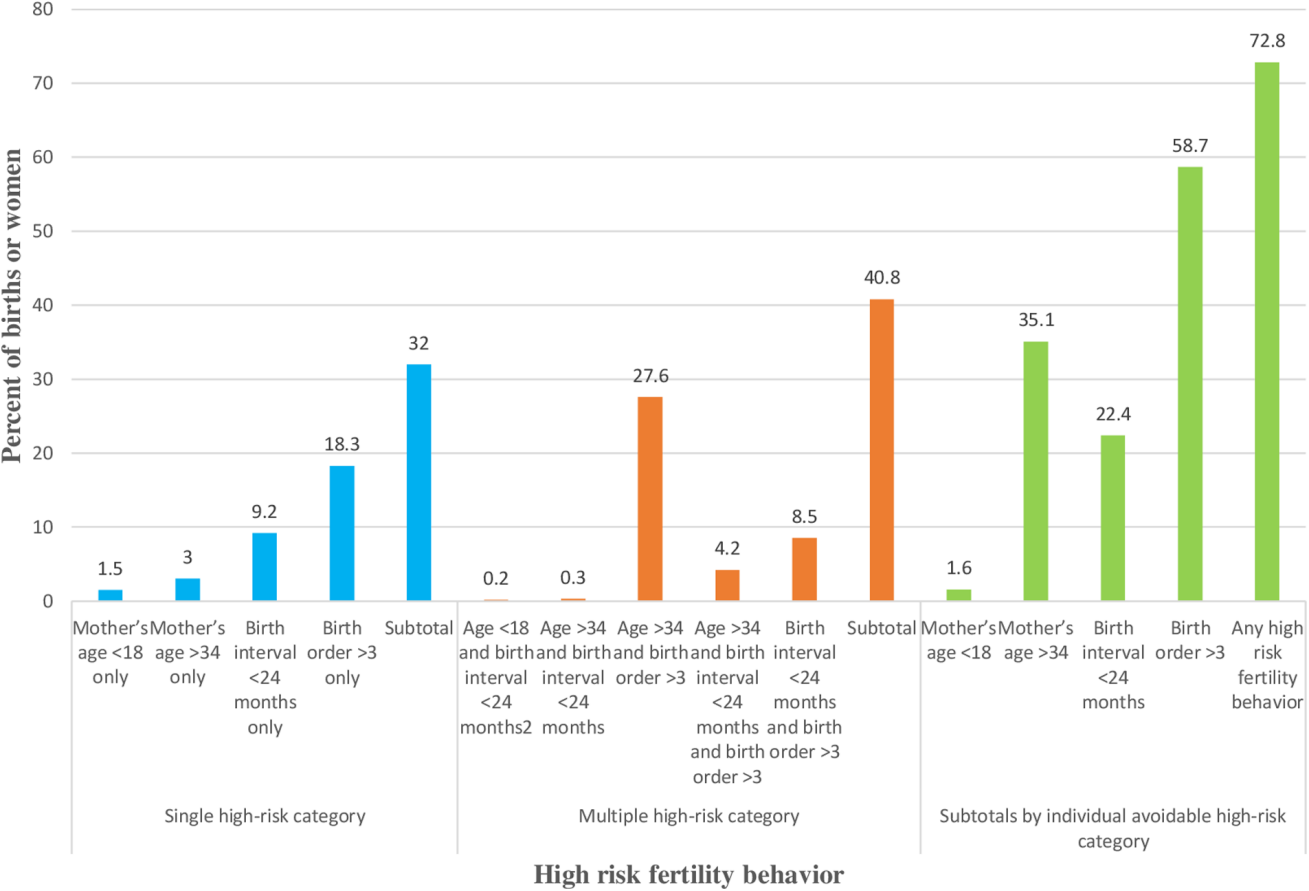
High-risk fertility behavior.

Nearly 73% (95% CI: 71.6%–73.9%) of the women had experienced high-risk fertility behavior. Of those women, around 32% had experienced a single high-risk fertility behavior and 40.8% had experienced multiple high-risk fertility behaviors. On the other hand, among those women who had experienced high-risk fertility behavior, 58.7% had more than three children; 22.4% had a short birth interval (less than 24 months); 35.1% were significantly old (over 34 years old); and 1.6% were significantly young (less than 18 years old) (Figure 1).

The results show that the women who had received no education (AOR = 4.31; 95% CI: 2.09, 8.89) or only primary education (AOR = 2.71; 95% CI: 1.63, 4.50) are more likely to experience high-risk fertility behavior than women with a higher level of education (Table 2). Moreover, every additional year of schooling reduces the odds of high-risk fertility behavior by 6% (AOR = 0.94; 95% CI: 0.89, 0.98). Reaching a secondary level of education has a significant negative impact on high-risk fertility behavior. Access to TV or radio is related to a 35% decline (AOR = 0.65; 95% CI: 0.44, 0.95) relative to women with no access to TV or radio. Women from the regions of Oromia (AOR = 1.45; 95% CI: 1.06, 1.97), Somali (AOR = 1.51; 95% CI: 1.05, 2.16), Gambella (AOR = 1.43; 95% CI: 1.03, 1.99), and Harari (AOR = 1.51; 95% CI: 1.06, 2.15) have a higher risk of high-risk fertility behavior than women from Addis Ababa.

**Table 2:** Bivariate and multivariable logistic regression analysis of high-risk fertility behavior.

	COR	95% C.I for COR	AOR	95% C.I for AOR
**Region**				
Tigray	2.028	1.545, 2.661	.952	.638, 1.420
Afar	2.786	2.110, 3.677	1.214	.739, 1.994
Amhara	2.579	1.985, 3.351	.821	.529, 1.274
Oromia	2.993	2.309, 3.881	1.447*	1.061, 1.974
Somali	4.861	3.571, 6.615	1.507*	1.052, 2.159
Benishangul-gumuz	2.747	2.088, 3.615	1.310	.864, 1.986
SNNPR	3.382	2.599, 4.402	1.288	.831, 1.996
Gambela	2.653	2.019, 3.487	1.428*	1.025, 1.990
Harari	2.195	1.665, 2.893	1.505*	1.055, 2.148
Dire Dawa	2.013	1.528, 2.652	1.293	.904, 1.849
Addis Ababa	1		1	
**Residence**				
Urban	.493	.438,.554	.989	.754, 1.297
Rural	1		1	
**Highest education level**				
No education	7.521	5.893, 9.599	4.307**	2.086. 8.889
Primary	2.422	1.893, 3.099	2.705**	1.625, 4.501
Secondary	1.362	1.022, 1.814	1.296	.967, 1.739
Higher	1		1	
**Access to TV or Radio**				
Yes	.459	.287,.735	.651*	.444,.954
No	1		1	
**Head of Household**				
Husband/Other	.980	.866, 1.110	.978	.790, 1.210
Respondent	1		1	
**Wealth quintile**				
Poorest	2.874	2.470, 3.346	1.045	.686, 1.592
Poorer	2.616	2.202, 3.108	1.316	.876, 1.977
Middle	2.240	1.884, 2.663	1.276	.859, 1.895
Richer	1.986	1.674, 2.356	1.240	.859, 1.789
Richest	1		1	
**Marital status**				
Single	.609	.355, 1.046	.901	.455, 1.784
Married	.844	.712. 1.000	.799	.606, 1.053
Others	1		1	
**Religion**				
Orthodox	.330	.204,.534	.316**	.158,.629
Protestant	.442	.272,.720	.367**	.187,.718
Muslim	.474	.293,.765	.275**	.137,.550
Others	1		1	
**Contraceptive use**				
Yes	0.525	.469,.589	0.739**	0.622, 0.879
No	1		1	
**Contraceptive knowledge**				
Knows modern methods	.803	.738,.873	.799*	.666,.959
Knows no methods	1		1	
**Highest year of education**	.945	.922,.970	.938*	.890,.988
**Goodness of fit test**				
**Hosmer and Lemeshow Test**			10.02, *p*-value = 0.264	
**Likelihood Ratio Test**			171.37, *p*-value < 0.001	

* Significant *p*-value < 0.05.

** Significant *p*-value < 0.01.

The findings in Table 2 confirm that women's knowledge and use of modern contraceptive methods reduce their risk of high-risk fertility behavior. The use of modern contraception reduces the chances of high-risk fertility behavior by 26% (AOR = 0.74; 95% CI: 0.622, 0.879), and knowledge of modern contraception methods is associated with a 20% (AOR = 0.80; 95% CI: 0.66, 0.96) reduction of the risk of HRFB. Certain religions, including Orthodox Christianity, Protestantism, and Islam, are associated with a lower chance of HRFB compared to Catholicism and other religions (*p*-value = 0.01).

**Figure 2:**
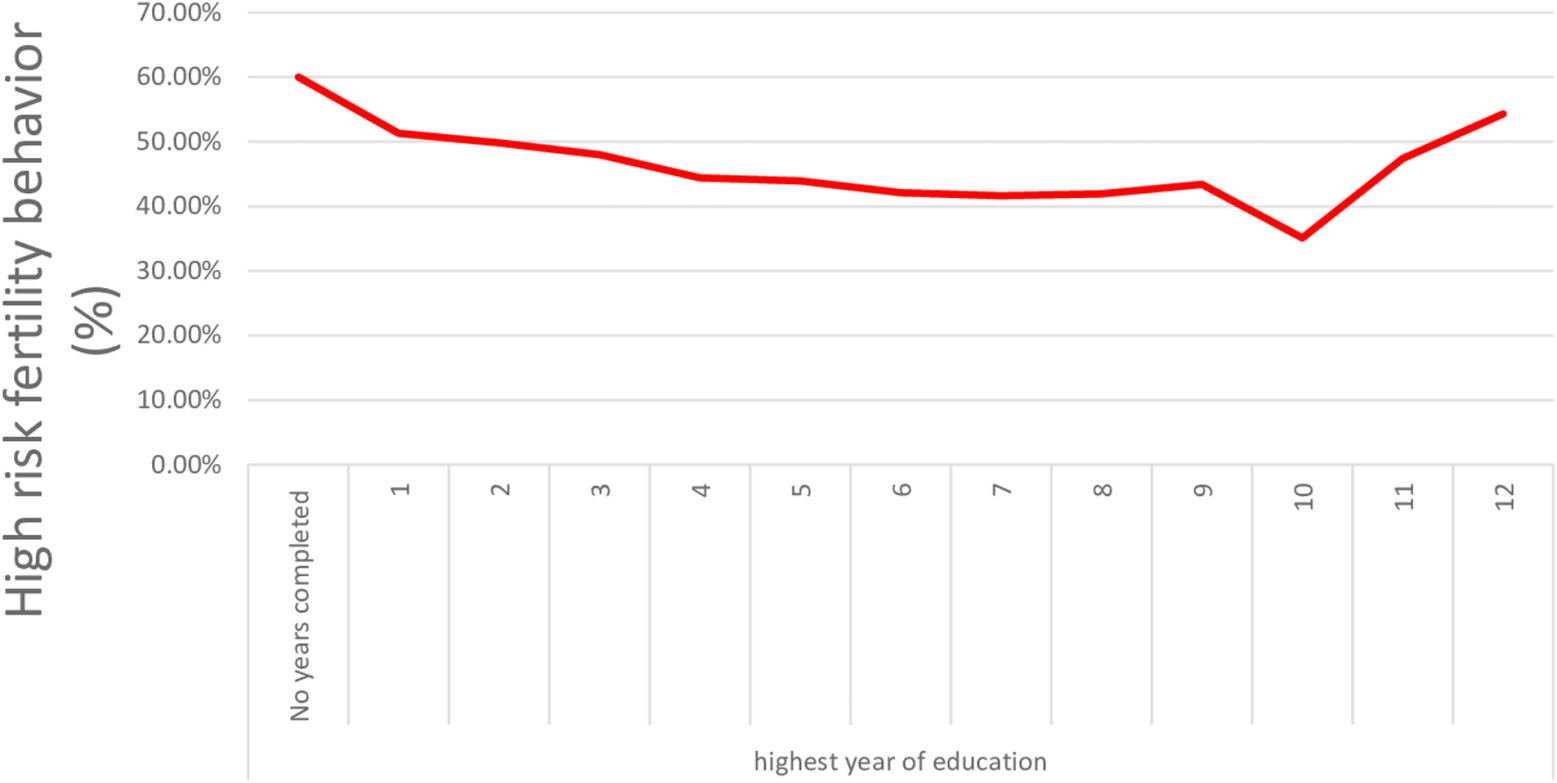
High-risk fertility behavior in Ethiopian women declines as women educational level increases.

Plotting the years of maternal education vs. the proportion of high-risk fertility allowed for a more in-depth analysis of the impact of women's education (Figure 2). The graph demonstrates that from 1st grade to 10th grade, as maternal education improves, the prevalence of high-risk fertility sharply decreases. After 10th grade, the prevalence of HRFB slightly rises (Figure 2).

## Discussion

This study analyzes the factors associated with high-risk fertility behavior using the 2019 EMDHS. This paper highlights the proportion of women with high-risk fertility behaviors that fall into either a single high-risk category, multiple high-risk categories, or any high-risk category in Ethiopia. This study found that 73% of women who had given birth in the previous 5 years were at high risk of high-risk fertility behaviors, of which, 32% were in the single-high-risk category and 40.8% were in the multiple-high-risk category. Higher birth order, more advanced age at birth, and a short birth interval are the common HRFBs. This is slightly higher than a study conducted in Bangladesh (67.7%) (34) and a pooled study in East Africa (57.6%) (35). This high prevalence of HRFB in Ethiopia may be due to the difference in child marriage practice, the unmet need for family planning, and harmful traditional practices. On the contrary, the prevalence of the single-high-risk category is lower than in a report from Chad (82.86%) (36).

The common stable predictors of high-risk fertility for women are a lower level of education, the lowest level of schooling, no access to TV or radio, not using contraception, and poor/no knowledge of modern contraceptive methods. Women's education is a significant predictor of high-risk fertility in Ethiopia (Table 2). We found that uneducated women are 4.3 times more likely to experience high-risk fertility compared to women who have a higher level of education. Higher levels of education are typically correlated with older marriage-age populations. Thus, enhancing women's educational levels has the potential to address the issue of early marriage and parenthood. Educated women are more likely to utilize contraception for spacing and limiting births and to engage in improved health-seeking behavior. Women with higher levels of education exhibit a lower propensity to engage in high-risk fertility compared to individuals who are uneducated. Other studies have reported reduced odds of HRFB in women with a higher level of education in Ethiopia (37), Bangladesh (34), India, Bangladesh, Nepal (38), Nigeria (39), and the East African regions (35). This exaggerated odds ratio of high-risk fertility among uneducated women could be due to the fact that educated women desire fewer children and have better control over childbearing, whereas illiterate women are physically more competent at giving birth. Educated women are also more likely to use contemporary birth control than illiterate women. Furthermore, education improves women's awareness of modern contraceptives and their capability to use new contraceptive methods. Goleen, S (30). considered all deliveries recorded in a woman's lifetime as a fertility measure in Egypt and found that advanced educational attainment has a considerable adjusted association with lower fertility in Egypt. A sub-Saharan African study found that women who use family planning and have completed at least primary school are less likely to engage in single and multiple high-risk fertility behaviors than their counterparts who do not use family planning and have had no formal education (36).

Contrary to our findings, using data from the 2016 EDHS, Tessema and Tamirat found that above secondary education does not significantly reduce the odds of high-risk fertility behavior in Ethiopia, whereas having primary or secondary education does significantly reduce it by 27%–29% (40). This demonstrates that a mother must attend school for a specific number of years before the protective effects of education are observed. Completing the 10th grade is associated with the lowest odds of high-risk fertility behavior in Ethiopia (Figure 2). According to an Egyptian study, the average number of births to uneducated women is 3.51, and the threshold for improving HRFB is at least intermediate education (30), while in Uganda, it is 8 years of schooling (41). Every additional year of maternal education is linked to a 6% decrease in the likelihood of HRFB (Table 2). In the Democratic Republic of Congo, with years of education between 6 and 7, the fertility rate is 6.6 deliveries per woman, and it is 4.4 in Malawi (25).

Only 6% of Ethiopian women have completed 8th grade, 1% have completed the fourth year of secondary school, and 6% have attained higher education. The median number of years of schooling is 2.5 years; thus, there is an urgent need to concentrate on girls' education (7). Secondary education or above is a protective factor for HRFB.

In this study, we discovered that women who use contraception have a lower risk of HRFB. This is in agreement with previous studies that found an association between contraception use and HRFB (42, 43). Women who use contraception have a 26% lower risk of HRFB than non-users (Table 2). Using contraceptives, women are more likely to delay births immediately after marriage, limit the number of births, and control the spacing of the preceding birth and current pregnancy than non-users. Secondly, knowledge of modern contraceptive methods must be considered along with contraceptive use. Knowledge of modern contraceptive methods reduces the odds of HRFB by 21%. Previous research has also found that contraception is a protective factor for HRFB. Women who use contraceptive methods have reduced odds of fertility in Uganda (41) and a 10% reduced risk of HRFB in the East African region (40). Non-use of contraception increases the odds of HRFB by 37% in Bangladesh (34), whereas knowledge of family planning lowers the odds of HRFB by 11% in East African countries (40). A conservative study based on DHS data from 45 countries conducted from 2006 to 2012 reported that 69% of women exhibit fertility-related risk, and 46% of non-pregnant women need a limiting method since they have had three births previously or are at least 40 years old. A lack of access to contraception is a problem for 21% of non-pregnant women due to their desires or risks (42).

The geographic region of residence of women is a significant predictor of HRFB in Ethiopia (Table 2). Women from the Oromia, Somali, Gambela, and Harari regions are at a higher risk of experiencing HRFB compared to Addis Ababa. This is congruent with previous studies in Ethiopia (34, 40, 44). This might be due to the differences in the most common harmful traditional practices in Ethiopia. Other than in Addis Ababa, early marriage is common, which has contributed significantly to early childbearing. In the 2019 EMDHS, only 3.4% of women in the Somali region used modern contraceptives (7).

The results show that access to TV or radio and the religion of a woman are associated with HRFB (Table 3). In the 2019 EMDHS, only 40% of Ethiopian women had media access (7). Access to TV or radio reduces the odds of HRFB by 35% in Ethiopia, whereas access to media reduces the incidence of HRFB based on the number of children ever born by 39% in Nigeria (45). However, in a study from Bangladesh (34), no association was found between watching TV and high-risk fertility behavior, while another study in the East African region reported no significant association between media exposure and HRFB (40). Orthodox Christians, Muslims, and protestants have a lower risk of HRFB than others. Previous studies (34) have also found religion to be a significant predictor of HRFB. In contradiction, Muslim vs. non-Muslim differentials of fertility and family planning in India (46) found that Muslims are more likely to be at a higher risk of having HRFB, but the authors argued that Muslim women are less likely to use contraceptives.

**Table 3:** Distribution of high-risk fertility behavior by characteristics of women.

		HRFB birth order	HRFB birth interval	HRFB age at birth		Any HRFB
		More than 3	< 24 months	< 18 years	35 or higher	
Variables	Categories	*n* (%)	(%)	*n* (%)	*n* (%)	*n* (%)
**Region**	Tigray	228 (45.2)	48 (12.4)	33 (6.5)	191 (37.9)	305 (60.5)
	Afar	231 (46.2)	131 (32.0)	26 (5.2)	138 (27.6)	339 (67.8)
	Amhara	323 (50.2)	58 (11.2)	24 (3.7)	286 (44.5)	425 (66.1)
	Oromia	410 (57.9)	145 (24.4)	23 (3.2)	273 (38.6)	491 (69.4)
	Somali	284 (66.0)	151 (38.6)	7 (1.6)	144 (33.5)	338 (78.6)
	Benishangul-gumuz	279 (53.3)	75 (17.6)	24 (4.6)	193 (36.9)	353 (67.5)
	SNNPR	409 (59.3)	133 (21.9)	26 (3.8)	282 (40.9)	496 (71.9)
	Gambela	258 (48.8)	83 (20.0)	49 (9.3)	174 (32.9)	353 (66.7)
	Harari	188 (39.5)	89 (24.3)	23 (4.8)	157 (33.0)	297 (62.4)
	Dire Dawa	182 (38.8)	69 (20.4)	28 (6.0)	166 (35.4)	283 (60.3)
	Addis Ababa	47 (12.6)	43 (18.7)	20 (5.3)	116 (31.0)	161 (43.0)
**Residence**	Urban	463 (28.1)	222 (19.3)	100 (6.1)	546 (33.1)	888 (53.9)
	Rural	2,376 (56.6)	803 (22.7)	183 (4.4)	1,574 (37.5)	2,953 (70.3)
**Highest education level**	No education	2,117 (66.7)	677 (23.4)	72 (2.3)	1,523 (48.0)	2,516 (79.2)
	Primary	599 (33.1)	263 (20.5)	160 (8.8)	427 (23.6)	998 (55.1)
	Secondary	97 (18.3)	56 (17.8)	35 (6.6)	108 (20.4)	216 (40.8)
	Higher	26 (7.9)	29 (15.3)	16 (4.8)	62 (18.8)	111 (33.6)
**Educational attainment**	No education	2,117 (66.7)	677 (23.4)	72 (2.3)	1,523 (48.0)	2,516 (79.)%
	Incomplete primary	551 (35.4)	238 (21.1)	131 (8.4)	379 (24.3)	884 (56.8)
	Complete primary	48 (18.9)	25 (16.2)	29 (11.4)	48 (18.9)	114 (44.9)
	Incomplete secondary	81 (18.0)	48 (18.0)	33 (7.3)	71 (15.7)	175 (38.8)
	Complete secondary	16 (20.5)	8 (16.7)	2 (2.6)	37 (47.4)	41 (52.6)
	Higher	26 (7.9)	29 (15.3)	16 (4.8)	62 (18.8)	111 (33.6)
**Head of Household**	Husband/other	2,200 (50.5)	752 (21.1)	181 (4.2)	1,511 (34.7)	2,857 (65.6)
	Respondent	639 (42.9)	273 (24.6)	102 (6.8)	609 (40.9)	984 (66.0)
**Wealth index**	Poorest	922 (60.9)	385 (28.9)	53 (3.5)	524 (34.6)	1,128 (74.5)
	Poorer	571 (59.0)	180 (22.0)	50 (5.2)	370 (38.2)	703 (72.6)
	Middle	499 (55.9)	138 (18.3)	32 (3.6)	362 (40.5)	620 (69.4)
	Richer	458 (51.5)	137 (18.8)	48 (5.4)	364 (40.9)	594 (66.8)
	Richest	389 (24.6)	185 (17.7)	100 (6.3)	500 (31.6)	796 (50.3)
**Access to TV or Radio**	No	1,967 (56.6)	693 (23.7)	156 (4.5)	1,265 (36.4)	2,466 (70.9)
	Yes	854 (37.1)	319 (18.6)	121 (5.3)	833 (36.2)	1,338 (58.2)
**Marital status**	Single	10 (16.9)	4 (21.1)	18 (30.5)	14 (23.7)	34 (57.6)
	married/living together	2,554 (50.3)	901 (21.7)	196 (3.9)	1,770 (34.8)	3,318 (65.3)
	Others	275 (38.8)	120 (24.0)	69 (9.7)	336 (47.5)	489 (69.1)
**Religion**	Orthodox	837 (40.6)	220 (14.3)	108 (5.2)	827 (40.1)	1,246 (60.4)
	Protestant	609 (52.3)	199 (20.8)	64 (5.5)	423 (36.3)	782 (67.1)
	Muslim	1,323 (52.9)	577 (27.8)	105 (4.2)	820 (32.8)	1,716 (68.6)
	Others	70 (59.3)	29 (28.2)	6 (5.1)	50 (42.4)	97 (82.2)
**Contraceptive use**	No	2,102 (53.2)	775 (23.9)	190 (4.8)	1,599 (40.5)	2,787 (70.5)
	Yes	737 (38.9)	250 (17.4)	93 (4.9)	521 (27.5)	1,054 (55.7)
**Ever attended school**	No	2,117 (66.7)	677 (23.4)	72 (2.3)	1,523 (48.0)	2,516 (79.2)
	Yes	722 (27.0)	348 (19.5)	211 (7.9)	597 (22.4)	1,325 (49.6)
**Highest educational level**	Primary	599 (33.1)	263 (20.5)	160 (8.8)	427 (23.6)	998 (55.1)
	Secondary	97 (18.3)	56 (17.8)	35 (6.6)	108 (20.4)	216 (40.8)
	Technical/vocational	9 (6.5)	13 (16.7)	6 (4.3)	23 (16.5)	49 (35.3)
	Higher	17 (8.9)	16 (14.3)	10 (5.2)	39 (20.4)	62 (32.5)
**Knowledge of modern contraceptive methods**	Knows no methods	239 (64.2)	104 (31.5)	11 (3.0)	157 (42.2)	290 (78.0)
	knows modern methods	2,592 (47.4)	921 (21.2)	272 (5.0)	1,959 (35.9)	3,542 (64.8)

The main strength of this study is that nationally representative data were used, which allows a broad view of results. In every analysis, sample weights were used. This study used the 2019 EMDHS. The DHS relies on self-reported data, which is prone to recall bias. Furthermore, the evaluation of women's autonomy, pregnancy intention, and desire for the child was not included because it was missing.

## Conclusions

No education, primary education only, and being residents of the Somali and Gambela regions are factors that significantly increase the risk of HRFB. However, years of schooling, knowledge and usage of modern contraceptives, and being an Orthodox Christian or Muslim reduce the risk of HRFB in Ethiopia. All strategies intended to lower maternal and newborn mortalities by reducing the risk of HRFB should contain important components such as educating and encouraging women to use modern contraceptives to prevent undesired pregnancies, limiting the number of births, and spacing their births. This highlights the need for laws to prevent young marriages and improved community support for reproductive healthcare, with an emphasis on the use of modern contraceptives for ideal childbearing.

## Data Availability

The original contributions presented in the study are included in the article/Supplementary Material, further inquiries can be directed to the corresponding author.
